# Single-Cell Analysis Revealed the Role of CD8^+^ Effector T Cells in Preventing Cardioprotective Macrophage Differentiation in the Early Phase of Heart Failure

**DOI:** 10.3389/fimmu.2021.763647

**Published:** 2021-10-20

**Authors:** Kyoko Komai, Minako Ito, Seitaro Nomura, Shigeyuki Shichino, Manami Katoh, Shintaro Yamada, Toshiyuki Ko, Mana Iizuka-Koga, Hiroko Nakatsukasa, Akihiko Yoshimura

**Affiliations:** ^1^ Department of Microbiology and Immunology, Keio University School of Medicine, Tokyo, Japan; ^2^ Division of Allergy and Immunology, Medical Institute of Bioregulation, Kyushu University, Fukuoka, Japan; ^3^ Department of Cardiovascular Medicine, Graduate School of Medicine, The University of Tokyo, Tokyo, Japan; ^4^ Division of Molecular Regulation of Inflammatory and Immune Diseases, Research Institute for Biomedical Sciences, Tokyo University of Science, Noda, Japan

**Keywords:** macrophages, heart failure, CD8^+^T cells, single-cell analysis, tissue repair

## Abstract

Heart failure is a complex clinical syndrome characterized by insufficient cardiac function. Heart-resident and infiltrated macrophages have been shown to play important roles in the cardiac remodeling that occurs in response to cardiac pressure overload. However, the possible roles of T cells in this process, have not been well characterized. Here we show that T cell depletion conferred late-stage heart protection but induced cardioprotective hypertrophy at an early stage of heart failure caused by cardiac pressure overload. Single-cell RNA sequencing analysis revealed that CD8^+^T cell depletion induced cardioprotective hypertrophy characterized with the expression of mitochondrial genes and growth factor receptor genes. CD8^+^T cells regulated the conversion of both cardiac-resident macrophages and infiltrated macrophages into cardioprotective macrophages expressing growth factor genes such as *Areg*, *Osm*, and *Igf1*, which have been shown to be essential for the myocardial adaptive response after cardiac pressure overload. Our results demonstrate a dynamic interplay between cardiac CD8^+^T cells and macrophages that is necessary for adaptation to cardiac stress, highlighting the homeostatic functions of resident and infiltrated macrophages in the heart.

## Introduction

Heart failure is one of the diseases for which few treatments exist. There are two main causes of heart failure: ischemic and non-ischemic. In the non-ischemic type, hypertension causes pressure overload hypertrophy of the heart, which eventually leads to heart failure. Myocardial hypertrophy is a morphological adaptive response to chronic work overload imposed on the heart. The relationship between myocardial hypertrophy and remodeling and immune cells, especially macrophages, has been well studied using a murine pressure overload heart failure model with transverse aorta constriction (TAC), which is a well-established model of pressure overload-induced cardiac hypertrophy and failure in mice (1).

Macrophages are the major immune cell type that is constitutively present within the myocardium. However, little is currently known about the origin and function of the cardiac macrophages that are involved in cardiac hypertrophy and heart failure. Although cardiac macrophages have traditionally been thought to be derived from circulating monocytes, recent studies have shown that cardiac-resident macrophages are derived from fetal tissues and are maintained, at least in part, through self-renewal (2). In addition, aging and pathological stimuli such as angiotensin II or myocardial infarction promote the accumulation of monocyte-derived macrophages (3, 4). Cardiac macrophages can be both protective and harmful in the context of myocardial infarction (5). It has become clear that the functions of macrophages are so diverse that neither their pathogenesis nor their therapeutic effects can be judged in a single or broad M1/M2 category. For example, cardiac tissue macrophages are known to be involved not only in wound healing during tissue injury (6), but also in promoting electrical atrioventricular conduction through connexin 43 (7). It has been reported that cardiac tissue macrophages are also involved in maintaining mitochondrial homeostasis (8), and that Amphiregulin (AREG), which is produced by Ly6c^low^ cardiac macrophages, is important for compensatory cardiac hypertrophy in a murine pressure-overload heart failure model (9).

In contrast, the role of T cells in orchestrating immune responses remains unclear in the context of heart failure. Although T cells have been detected in the heart following coronary artery occlusion in rodents, the roles of these cardiac T cells in heart failure are controversial (10, 11). A recent study has shown that, following acute heart infarction in mice, CD8^+^T cells recruited in the ischemic heart promoted cardiomyocyte death through the local release of Granzyme B, leading to enhanced myocardial inflammation, tissue injury, and deterioration of myocardial function (12). Previous reports have suggested that CD4^+^T cells are directly involved in the progression from hypertrophy to heart failure (13–15), yet the precise mechanism by which T cells promote heart failure remain to be clarified.

　In this study, we investigated the dynamics of immune cells and cardiac muscle cells in a murine TAC model using single-cell RNA sequencing (scRNAseq) techniques. We found that cardiac macrophages, which play an important role in compensatory cardiac hypertrophy, were affected by T cells, especially CD8^+^T cells. Depletion of CD8^+^T cells led to an increase in tissue macrophages, and eventually alleviated heart failure by promoting cardioprotective hypertrophy. This is the first report showing a relationship between cardiac macrophages and T cells, a finding that could lead to future therapeutic approaches.

## Results

### T Cell Depletion Suppresses Heart Failure at Late Phase but Promotes Hypertrophy at Early Phase in a TAC Model

**Figure 1:**
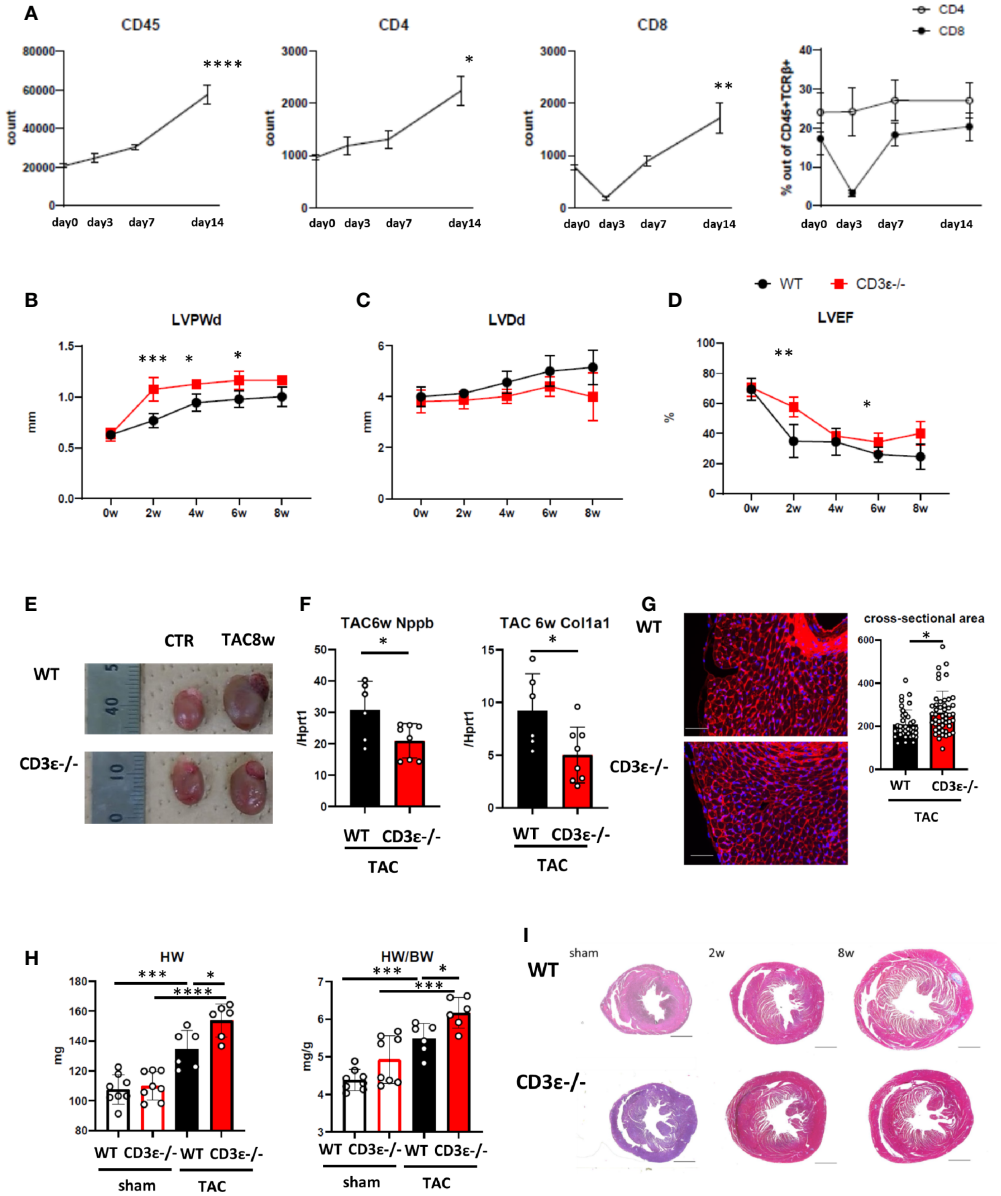
T cell depletion suppresses heart failure at late phase but promotes hypertrophy at early phase after TAC. **(A)** Changes in numbers of CD45^+^cells, the numbers, and the population of CD4^+^T and CD8^+^T cells gated on CD45^+^TCRβ^+^ cells isolated from heart after TAC. Day 0 represents the measurement before TAC. *P* values *vs.* day0. (n=4 in each point) **(B-D)** Echocardiography up to 8 weeks after TAC. 0 week indicates the measurement before TAC treatment. *P* values *vs.* WT. (WT n=7, CD3ϵ^-/-^ n=6 After the measurement at 2w, two mice in each group were excluded for single cell analysis.) **(E)** Gross appearance of heart 8 weeks after TAC. **(F)** mRNA (*Nppb* and *col1a1/Hprt1*) levels extracted from whole heart 6 weeks after TAC. (n=6-8) **(G)** WGA staining 2 weeks after TAC. The cross-sectional area of the myocardium was measured by extracting the myocardium with the nucleus at its center from each slide made from n=4 in each group. Scale bar: 50µm. **(H)** Heart weight and heart weight/body weight 2 weeks after TAC. (n=6-8) **(I)** Short-axis histology of heart two and 8 weeks after TAC (Masson’s trichrome staining). Scale bar: 1mm. Data points are individual mice in one of two individual experiments, except for B-D. *p* values were determined by two-tailed Student’s t-test or one-way analysis of variance (ANOVA). Data are means ± SD. *p < 0.05, **p < 0.01, ***p < 0.001 and ****p < 0.0001.

As previously reported (13), the number of T cells in the heart increased after TAC (**Figure 1A** and **Supplementary Figures 1A–D**). Our preliminary data suggest that most CD8^+^T cells in the heart before TAC showed CD44^+^CD62L^high^ memory phenotype, and CD44^+^CD62L^low^ effector fraction slightly increased after TAC (data not shown). To determine the effects of T cells on heart failure, we performed TAC on T cell-deficient CD3ϵ^-/-^ mice as a model for heart failure. T cell deficiency can affect macrophage functions. Echocardiography showed an increase in left ventricular posterior wall thickness at diastole (LVPWd) 2 to 4 weeks after TAC, followed by a delayed increase in left ventricular diastolic diameter (LVDd) (**Figures 1B, C**). Left ventricular ejection fraction (LVEF) decreased 2 weeks after TAC and continued to decline (**Figure 1D**). In contrast, although CD3ϵ^-/-^ mice showed a rapid increase in LVPWd 2 weeks after TAC, LVDd was smaller in CD3ϵ^-/-^ mice than in WT mice, and LVEF was maintained at high levels in CD3ϵ^-/-^ mice compared with WT mice (**Figures 1B–D**), indicating that cardiac dilatation was suppressed and cardiac output was effectively maintained in CD3ϵ^-/-^ mice. Eight weeks after TAC, LVDd was strongly suppressed in CD3ϵ^-/-^ mice, and heart size was smaller in CD3ϵ^-/-^ mice than in WT mice (**Figures 1C, E**). Furthermore, CD3ϵ^-/-^ mice had lower mRNA levels of *Nppb*, which encodes brain natriuretic peptide (BNP), a marker of heart failure, as well as lower levels of *Col1a1*, a fibrosis marker, compared to WT mice (**Figure 1F**). These data indicate that in the genetic absence of T cells protects the heart from heart failure induced by pressure overload.

However, we noticed that the LVPWd was greater in CD3ϵ^-/-^ mice than in WT mice 2 weeks after TAC, indicating that the myocardium had thickened by the absence of T cells (**Figure 1B**). Histological analysis confirmed the cardiac hypertrophy is caused by cardiomyocyte enlargement in CD3ϵ^-/-^ mice (**Figure 1G**). Indeed, heart weight and wall thickness were consistently larger in CD3ϵ^-/-^ mice than in WT mice; this difference was especially marked 2 weeks after TAC (**Figures 1B, H**). The tendency of higher HW/BW in sham CD3ϵ^-/-^ mice is probably due to smaller BW of CD3ϵ^-/-^ mice (sham WT 24.56 ± 1.45g, sham CD3ϵ^-/-^ 22.46 ± 1.56g), though there was no difference in BW after TAC (TAC WT 24.50 ± 1.22g, TAC CD3ϵ^-/-^ 24.93 ± 1.48g). The hearts of CD3ϵ^-/-^ mice enlarged rapidly 2 weeks after TAC but did not enlarge progressively and did not show severe fibrosis 6 to 8weeks after TAC (**Figures 1F, I**). **Figure 1I** showed a representative viewing of the heart. We confirmed the there is no difference in collagen at mRNA levels before TAC (data not shown). This implies that heart hypertrophy does not always lead to heart failure; accordingly, we hypothesized that the cardiac hypertrophy seen in the CD3ϵ^-/-^ mice in the early stages after TAC is cardioprotective.

### CD8^+^T Cell Depletion Results in Cardioprotective Hypertrophy

**Figure 2:**
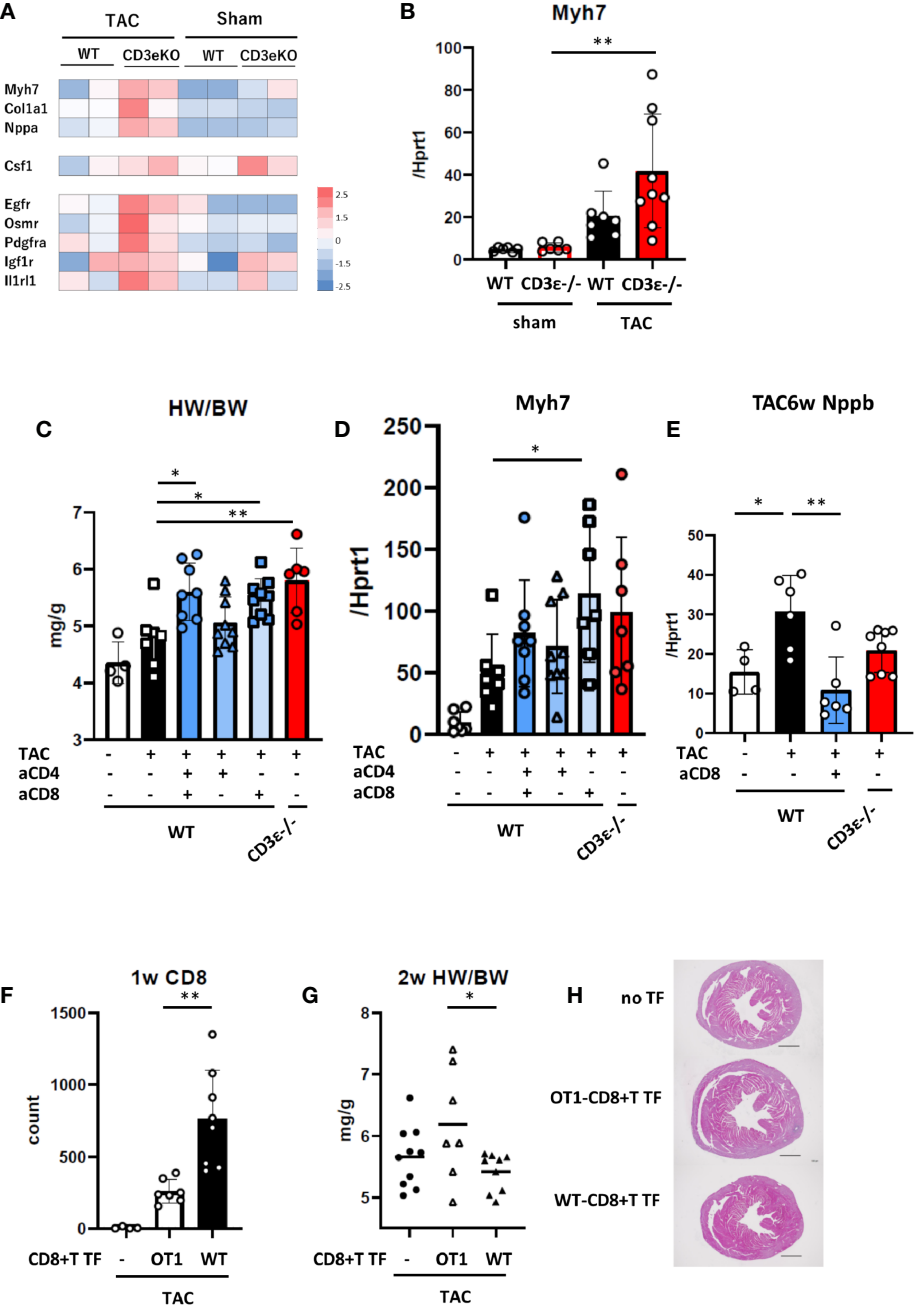
CD8^+^T cell depletion results in cardioprotective hypertrophy. **(A)** Expression of genes related to cardiac hypertrophy and tissue repair from whole RNAseq data of cardiomyocytes 2 weeks after TAC. (n=2) **(B)** mRNA (*Myh7/Hprt1*) extracted from whole heart 2 weeks after TAC determined by q-PCR. (n=6-9) **(C)** Heart weight/body weight of heart 2 weeks after TAC. (n=4-9) **(D)** mRNA (*Myh7/Hprt1*) extracted from whole heart 2 weeks after TAC determined by q-PCR. (n=6-9) **(E)** mRNA (*Nppb/Hprt1*) extracted from whole heart 6 weeks after TAC determined by q-PCR. (n=4-8) For CD4^+^ or CD8^+^T cell depletion, mice received an i.p. injection of anti-CD4 antibody or anti-CD8 antibody or vehicle (PBS) on day 0 and every three days until 2 weeks after TAC. **(F)** WT or OT-I-CD8^+^T cells were transferred (TF) into CD3ϵ^-/-^ mice five days prior to the TAC operation, then cell the number of CD8^+^T cells was counted in the heart 1 week after TAC. (n=4-8) **(G)** Heart weight/body weight 2 weeks after TAC in WT or OT-I-CD8^+^T cells-transferred (TF) mice. (n=7-10) **(H)** Short-axis histology of heart 2 weeks after TAC (H&E staining). Representative picture of samples. Scale bar: 1mm. Data points are individual mice in one of two or three individual experiments, except for RNAseq analysis*. p* values were determined by two-tailed Student’s t-test or one-way analysis of variance (ANOVA). Data are means ± SD. *p < 0.05, **p < 0.01.

To confirm the presence of the cardioprotective hypertrophy in the early stages after TAC, we performed RNAseq analysis on isolated cardiomyocytes 2 weeks after TAC treatment (**Figure 2A**). Sarcomeric cardiac β-myosin heavy chain gene (*Myh7)* has been shown to be associated with myocardial hypertrophy (16, 17). Natriuretic Peptide A (*Nppa*) has secreted by cardiac stretch stimulation, and collagen type I alpha 1 Chain (*Col1a1)* is a marker of subsequent fibrosis (13). The expression levels of *Myh7*, *Col1a1*, and *Nppa* were higher in CD3ϵ^-/-^ mice than in WT mice (**Figure 2A**), and we confirmed that TAC induced *Myh7* more intensively in CD3ϵ^-/-^ mice than in WT mice (**Figure 2B**).

The cardioprotective cytokine receptors *Egfr*, *Osmr*, *Pdgfra*, *Igf1r*, *Il1rl1* (receptors for EGF, OSM, PDGF, IGF-1, and IL-33, respectively) and macrophage colony stimulation factor (*Csf1*) were also higher in CD3ϵ^-/-^ mice than in WT mice (**Figure 2A**). These data further confirmed that cardioprotective hypertrophy occurs in CD3ϵ^-/-^ mice at an early stage after TAC.

Next, to determine which CD4^+^ or CD8^+^T cells are involved in cardioprotective hypertrophy, we depleted these cells using CD4- or CD8-specific antibodies. Antibodies to CD4 and CD8 were administered every three days for 2 weeks after TAC and heart weight and *Myh7* mRNA levels were measured (**Figures 2C, D** and **Supplementary Figures 2A, B**). Anti-CD4 antibody alone had little effect, while anti-CD8 antibody or a combination of anti-CD4 and anti-CD8 antibodies increased heart weight and promoted *Myh7* expression as observed in CD3ϵ^-/-^ mice. CD8^+^T cell depletion decreased the expression of *Nppb*, a heart failure marker (13), 6 weeks after TAC (**Figure 2E**).

We determined whether antigen recognition of CD8^+^T cells is required for cardioprotective hypertrophy through experiments with OT-I mice, in which most CD8^+^T cells recognize the OVA peptide/class I MHC complex. We transferred CD8^+^T cells isolated from the spleens of WT (WT-CD8^+^T) or OT-I (OT-I-CD8^+^T) mice into CD3ϵ^-/-^ mice, then performed TAC. One week after TAC, CD8^+^T cell infiltration into the heart was significantly higher in mice transferred with WT-CD8^+^T cells than in those transferred with OT-I-CD8^+^T cells (**Figure 2F** **Supplementary Figures 2C, D**). Heart weight at 2 weeks post-TAC was lower in mice transferred with WT-CD8^+^T cells than in those transferred with OT-I-CD8^+^T cells (**Figure 2G**). Histological analysis supports that WT-CD8^+^T cell transfer suppress cardiac hypertrophy, but not in OT-I-CD8^+^T transfer group (**Figure 2H**). Since OT-I-CD8^+^T cells and WT-CD8^+^T cells might infiltrate and proliferate in the heart differently, this experiment suggests a potential importance of antigen recognition of CD8^+^T cells.

These results indicate that the absence of CD8^+^T cells enhanced early cardioprotective hypertrophy induced by pressure overload.

### CD8^+^T Cell Depletion Promotes Early Changes in Cardioprotective Metabolic Gene Expression After TAC

**Figure 3:**
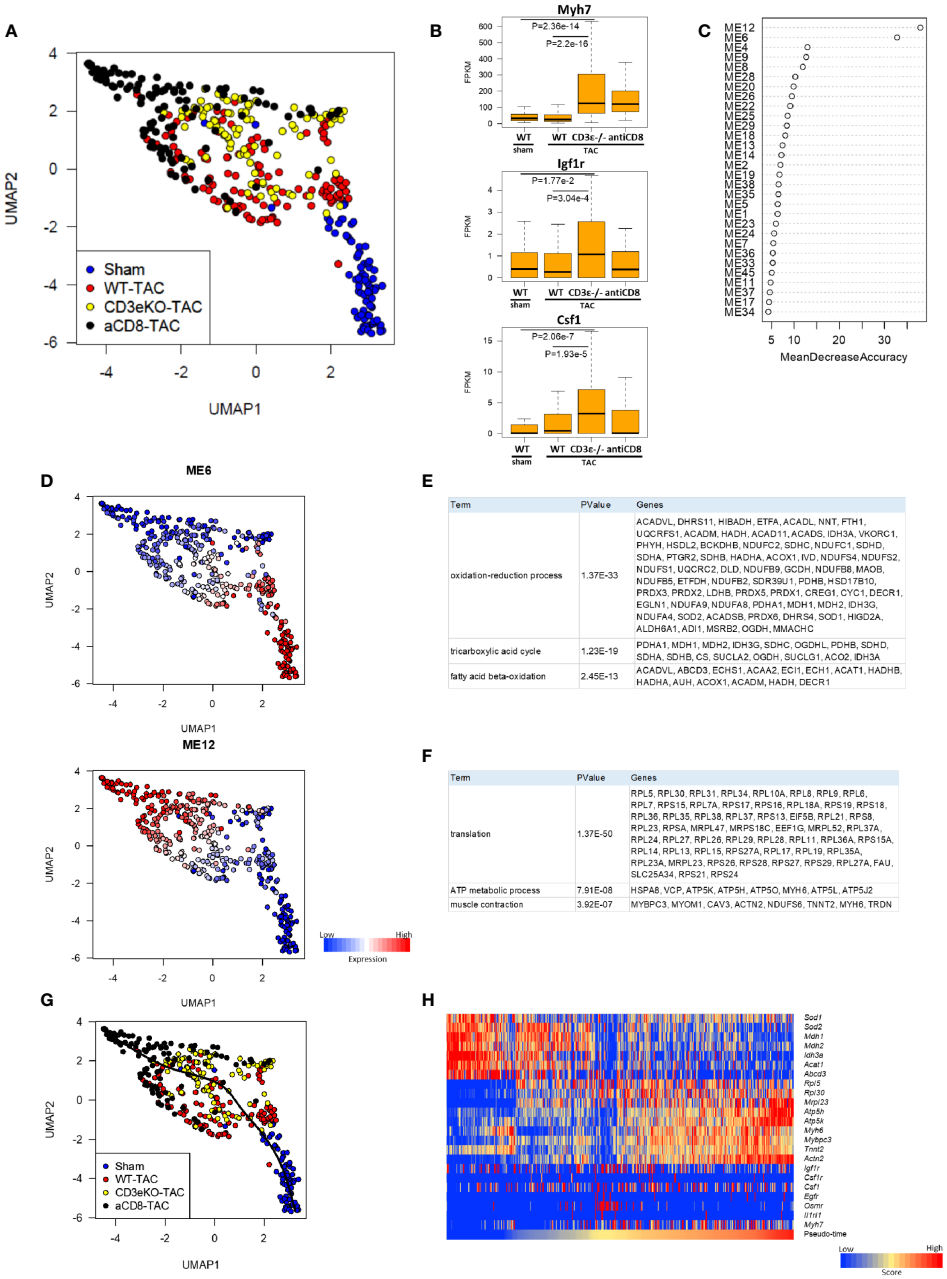
CD8^+^T cell depletion promotes early changes in cardioprotective metabolic gene expression after TAC. **(A)** UMAP plots of cardiomyocytes from sham, WT, CD3ϵ-/-, and CD8^+^T cell depleted mice at 2 weeks after TAC. Cells (dots) are colored for each group. scRNAseq analysis was performed as described in the Materials and Method section. **(B)** Box plots of representative expression of each gene in cardiomyocytes (*Myh7*; myocardial hypertrophy, *Igf1r*; growth factor receptor, *Csf1*; macrophage induction). Statistical differences were analyzed with a Wilcoxon rank sum test. **(C)** Mean decrease in accuracy (in order of decreasing accuracy from top to bottom) of each module eigengene (ME) as assigned by the random forest classifier. Random Forest algorithm was performed on 45 modules identified by weighted gene co-expression network analysis (WGCNA) restricted to 11,979 genes expressed at a fragments per kilobase of exon per million reads mapped (FPKM) value of ≥5 in at least 5 cardiomyocytes from a total of 21,859 genes. ME; the first principal component of the expression profile of genes belonging to the module. **(D)** UMAP plots colored by the expression of each module. **(E, F)** List of genes constituting M6 **(E)** and M12 **(F)**. **(G)** Single-cardiomyocyte trajectory on the UMAP plot reconstructed by Slingshot. The lower right part, where cardiomyocytes from sham mice were enriched, was defined as the starting point. **(H)** Heatmap showing expression of M6/12 genes and cardioprotective cytokine receptor genes in pseudo-time order.

Next, to define the mechanism underlying CD8^+^T cell depletion-mediated cardioprotective hypertrophy, we performed scRNAseq analysis of cardiomyocytes isolated from mice 2 weeks after TAC using Langendorff perfusion (16). We have previously shown that, during early hypertrophy, cardiomyocytes activate mitochondrial metabolism and protein synthesis genes, and that their expression levels are correlated with cell size (16). In the present study, principal component analysis (UMAP) revealed dramatic differences among WT mice, CD8^+^T cell-depleted mice, and CD3ϵ^-/-^ mice in gene expression in cardiomyocytes after TAC (**Figure 3A**). Expressions of the hypertrophy marker *Myh7* and the tissue repair-related genes *Igf1r* and *Csf1* were most evident in CD8^+^T cell-depleted mice and CD3ϵ^-/-^ mice compared with WT mice (**Figure 3B**), which is consistent with total RNAseq data (**Figure 2A**).

We used the Random Forests machine learning algorithm to extract modules that were significant for cell classification, and identified modules M6 and M12 as the gene modules that were most drastically different between WT mice and CD8^+^T cell-depleted or CD3ϵ^-/-^ mice (**Figures 3C, D** **)**. M6 was enriched for genes involved in the oxidation-reduction process, the TCA cycle, and fatty acid beta-oxidation, while M12 was enriched for genes involved in translation, ATP metabolism, and muscle contraction. The representative genes that form these modules are shown in **Figures 3E, F**. Cardiomyocytes of CD8^+^T cell-depleted mice and CD3ϵ^-/-^ mice showed lower M6 expression and higher M12 expression compared with those of WT mice. Pseudo-time analysis is a powerful tool for the transcriptional dynamics during the trajectory of cardiomyocyte remodeling as reported previously (16). The pseudo-time calculated by trajectory analysis confirmed that the M6/12 genes and cardioprotective cytokine receptor genes were sorted in pseud-time order (**Figures 3G, H**). Decrease in M6 genes suggest reduced oxidative stress and increase of M12 genes suggests anabolism and preservation of muscle functions. The resulting metabolic and translational signature in cardiomyocytes may be responsible for the observed myocardial protection and subsequent alleviation of heart failure.

### CD8^+^T Cell Depletion Modulates Macrophage Status

Macrophages have been shown to play essential roles in the pathology of TAC. For example, bone marrow-derived CD72^hi^ macrophages and CD68^hi^ inflammatory macrophages have been shown to promote cardiac injury (18, 19), while an increased M2-type macrophage count protects against heart injury (20). T-cell depletion promotes the expression of *Csf1*, a macrophage colony stimulating factor (M-CSF) that usually promotes M2 type macrophage differentiation (21) (**Figures 2A**, **3B**). Therefore, we hypothesized that the presence of CD8^+^T cells modifies macrophage status which leads to cardioprotective hypertrophy.

**Figure 4:**
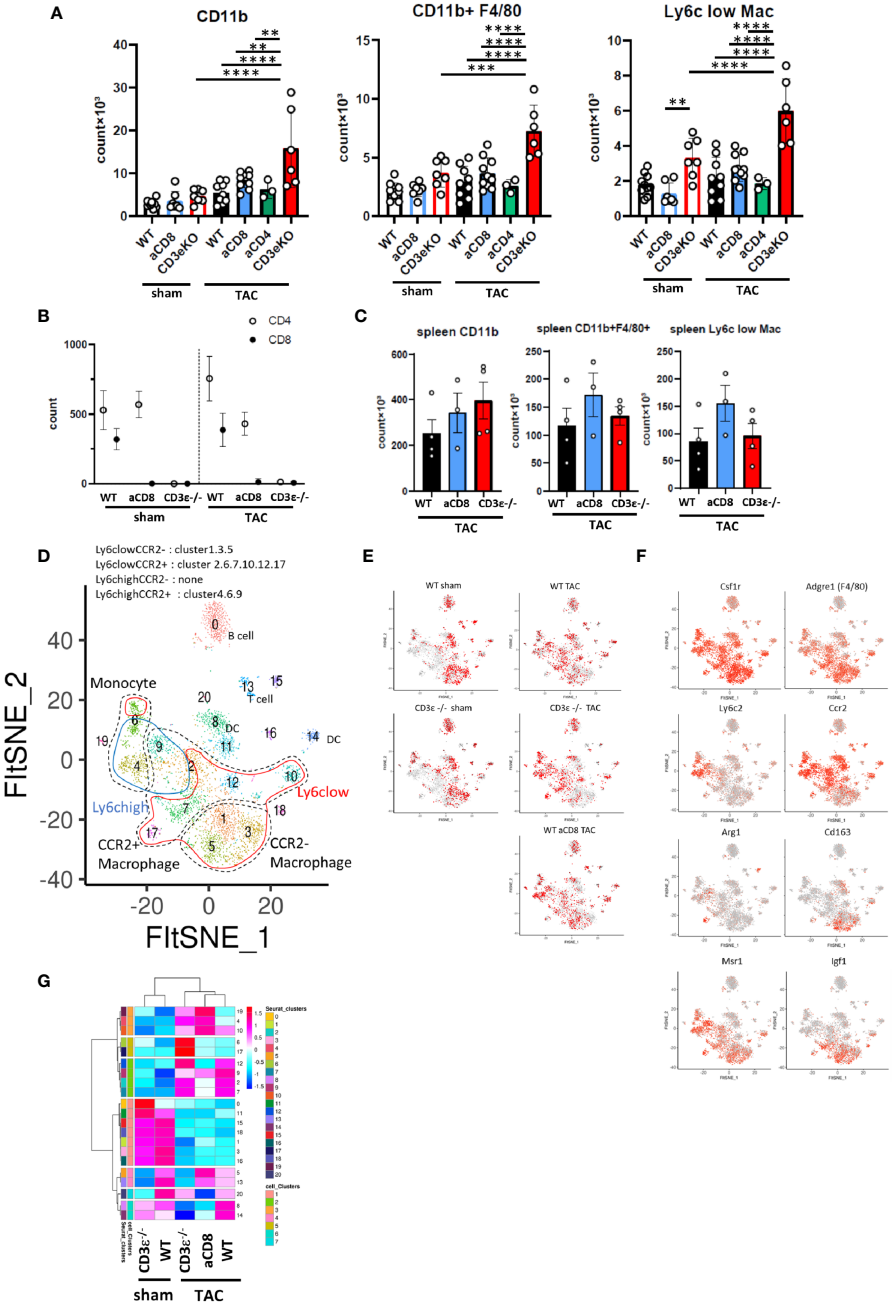
CD8^+^T cell depletion modulates macrophage status. **(A)** Numbers of CD11b^+^ cells, F4/80^+^ macrophages (CD11b^+^F4/80^+^), and Ly6c^low^ macrophages (CD11b^+^F4/80^+^Ly6c^low^) gated on CD45^+^ FVD^-^ CD3ϵ^-^ CD19^-^ Ly6g^-^ cells isolated from heart 1 week after sham/TAC operation. (n=6-10) For CD4^+^ or CD8^+^T cell depletion, mice received an i.p. injection of anti-CD4 antibody or anti-CD8 antibody on day 0 and every three days until 1 week after sham/TAC. **(B)** Numbers of CD4^+^T and CD8^+^T cells gated on CD45^+^TCRβ^+^ cells isolated from heart after sham/TAC. (n=4 per group) **(C)** Numbers of CD11b^+^ cells, F4/80^+^ macrophages, and Ly6c^low^ macrophages isolated from spleen 1 week after TAC operation. (n=3-4) **(D)** scRNAseq FltSNE display of cardiac CD45-positive cells 1 week after sham/TAC operation. Classical macrophage/monocyte fractions corresponding to clusters are shown. **(E)** Cell distribution under each condition (cell numbers are aligned). **(F)** Gene expressions on the FltSNE plot. **(G)** Cluster compositions of each group. After calculating the % of total cells of each cluster in each sample, the cell compositions of each cluster were z-scaled, clustered based on Pearson’s correlation coefficient using hclust (according to the Ward method) and visualized using pheatmap. Data points are individual mice in one of two or three individual experiments, except for single cell analysis. *p* values were determined by two-tailed Student’s t-test or one-way analysis of variance (ANOVA). Data are means ± SD. **p < 0.01, ***p < 0.001 and ****p < 0.0001.

To verify this hypothesis, we first performed FCM analysis of cardiac inflammatory cells 1 week after TAC, when cardiac hypertrophy was not very evident. At this time point, TAC increased the number of CD11b^+^ cells and F4/80^+^ macrophages as well as Ly6c^low^ cardiac tissue-resident macrophages, and CD3ϵ^-/-^ mice showed much higher numbers of these cells compared to WT mice (**Figure 4A** and **Supplementary Figures 1E, F**). A similar tendency was observed in CD8^+^T cell-depleted mice, but not in CD4^+^T cell-depleted mice. The deletion of CD8^+^T cells had no effect on the number of CD4^+^T cells (**Figure 4B**). The number of resident and infiltrated macrophages did not change in the spleens of WT, CD8^+^T cell-depleted and CD3ϵ^-/-^ mice after TAC (**Figure 4C**), suggesting that T cell deficiency increased monocytes and macrophages in the heart in a tissue-dependent manner.

To analyze the differences in the monocyte and macrophage fractions in detail, we performed scRNAseq analysis of the CD45^+^ mononuclear cells found in the heart. We identified macrophage/monocyte by the expression of *Csf1r*, *Lyz1, and Lyz2*. Among them, cluster 4 and 6 were identified as monocytes with low expression of *Adgre1* (F4/80) and *Cd68*. In particular, cluster 6 has high expression of *Cd300e*.　Cluster1, 3, and 5 were identified as CCR2^-^ macrophage and others are CCR2^+^ macrophages (cluster 2, 7, 9, 10, 12, and 17) (**Figures 4D, F** and **Supplementary Table 2**). At steady-state levels, the majority of monocytes/macrophages in the heart were Ly6c^low^CCR2^-^ resident macrophages (clusters 1, 3, and 5) in both WT and CD3ϵ^-/-^ mice (**Figures 4D, E, G**). One week after TAC, these Ly6c^low^CCR2^-^ clusters drastically decreased while Ly6c^low^ CCR2^+^ clusters 2 and 7 increased (**Figures 4D, E, G**). It is notable that TAC increased clusters 4 and 6, which represent CD11b^+^F4/80^-^ monocytes, in both CD3ϵ^-/-^ mice and CD8^+^T cell-depleted mice compared with WT mice (**Figure 4G**). Cluster 17, containing Ly6c^low^CCR2^+^ macrophages, was specifically increased in CD3ϵ^-/-^ mice and strongly expressed *Arg1*, a cardioprotective cytokine (9) (**Figure 4F**). Cluster 5, which also contained Ly6c^low^CCR2^+^ macrophages, was increased in CD8^+^T cell-depleted TAC mice, but not in CD3ϵ^-/-^ mice, compared with WT TAC mice; these cells strongly expressed *Cd163*, a M2 type macrophage marker (**Figure 4F**). Although there are some differences in the subclusters of macrophages between CD8^+^T cell-depleted mice and CD3ϵ^-/-^ mice, the overall picture suggests the depletion of CD8^+^T cells facilitated the infiltration of monocytes into the heart and the expansion of Ly6c^low^CCR2^+^ macrophages.

Ly6c^high^CCR2^+^ cells are usually bone marrow-derived inflammatory macrophages, but here they showed both M1- and M2-like phenotypes expressing *Nos1, Trl2/4, Arg1*, and *Msr1*, probably because their seven days of residence in the heart modulated their phenotypes, in keeping with our previous finding that Ly6c^high^CCR2^+^ cells can convert from inflammatory cells into repairing M2-like cells expressing Msr1 and IGF-1 in the brain from day 3 to day 6 after ischemic brain injury (22). Because clusters 4, and 9 also highly expressed *Msr1* (**Figure 4F** **)**, these data suggest that a lack of CD8^+^T cells increases the numbers of both resident and infiltrated macrophages and monocytes and also promotes phenotypic conversion into M2-type like cells.

### Cardiac Macrophage Differentiation Defined by RNA Velocity Analysis

**Figure 5:**
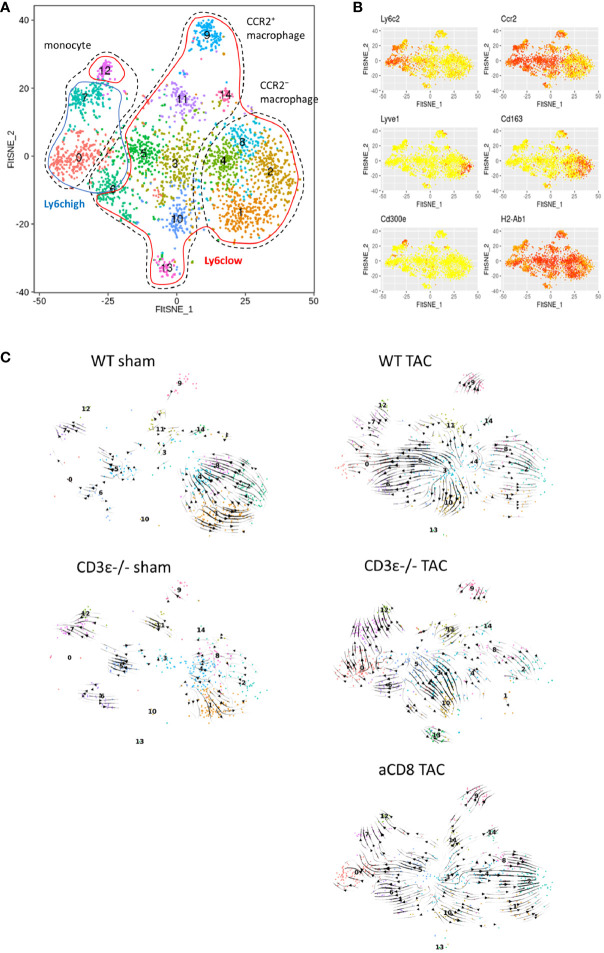
Cardiac macrophage differentiation defined by RNA velocity analysis. **(A)** scRNAseq FltSNE display of cardiac monocyte/macrophage subpopulation 1 week after sham/TAC operation. **(B)** Indicated gene expressions on the FltSNE plot. **(C)** RNA velocity analysis of cardiac monocyte/macrophage clusters sham and 1 week after operation in WT, CD3ϵ^-/-^, and CD8^+^T cell depleted TAC mice. Analysis was against monocyte/macrophage clusters (CD45^+^ cell clusters 1 to 7, 9, 10, 12, and 17 in **Figure 4C**).

To further investigate the difference in monocyte/macrophage differentiation between WT, CD3ϵ^-/-^, and CD8^+^T cell-depleted TAC mice, we performed RNA velocity analysis against monocyte/macrophage subclusters (**Figures 5A, B**). In sham mice, the direction of differentiation of resident macrophages was similar among WT and CD3ϵ^-/-^ mice. In TAC mice, the direction of Ly6c^hi^ monocytes (clusters 0, 7, and 12) was similar among WT and CD3ϵ^-/-^ mice (**Figure 5C**). There seems to be two types of monocyte-like cells (clusters 3 and 5), which are probably a different population from the peripheral blood monocyte (cluster 7: Ly6C^high^, 12: Ly6C^low^). There was a direction from cluster 3 to cluster 5 and 0 or cluster 10, with a particularly strong direction to cluster 5 and 0 in WT TAC mice. Whereas, in CD3ϵ^-/-^ TAC mice, there was strong direction from cluster 5 to cluster 3 and 10. In CD3ϵ-/- and CD8^+^T cell-depleted TAC mice, the direction from cluster 3 to cluster 4 was also observed. Especially in CD8^+^T cell-depleted TAC mice, there was a strong supply flow from cluster 3 to resident macrophages (cluster 1, 2, 4, and 8). In addition, our velocity analysis indicated that CD3ϵ^-/-^ TAC mice-specific cluster 13 originated from cluster 10 (**Figure 5C**). These results showed a pathway from cluster 3 and 5 (CCR2^+^monocyte-like cells) to cluster 10 and 13 (resident macrophages) and this flow was facilitated in the absence of CD8^+^T cells.

### CD8^+^T Cells Affect Expression Patterns of Macrophage Repairing Factors

**Figure 6:**
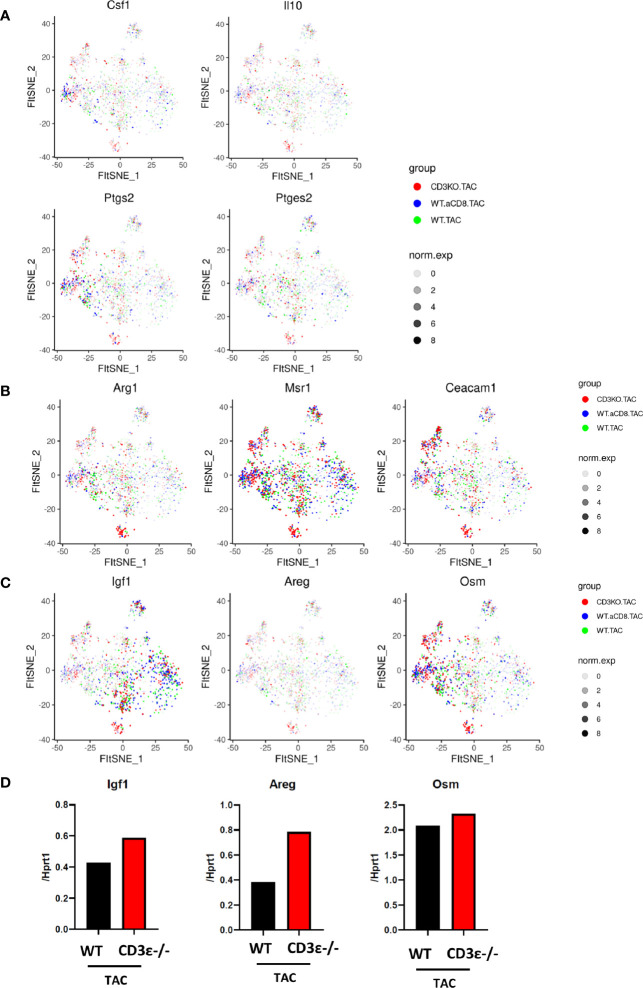
CD8^+^T cells affect expression patterns of macrophage repairing factors. **(A, B)** Gene expression of CD45-positive cells 1 week after TAC in monocyte/macrophage clusters. **(C)** Expression of *Igf1, Areg*, and *Osm* 1 week after TAC (red: CD3ϵ^-/-^KO TAC; blue: CD8^+^T cell depleted TAC; green: WT TAC) in monocyte/macrophage clusters. norm.exp: normalized expression [ln(x+1)]. **(D)** mRNA (*Igf1, Areg*, and *Osm/Hprt1*) of CD45^+^ (CD3ϵ^-^ CD19^-^ Ly6g^-^) cells collected from 4-10 pooled hearts 1 week after TAC.

Next, we compared the characteristics of cardiac monocytes/macrophages whose numbers were increased by TAC. As shown in **Figures 6A, B**, clusters expressing *Csf1, Il10, Ptgs2, Areg*, and *Msr1* were increased in CD3ϵ^-/-^ mice and CD8^+^T cell-depleted mice compared with WT mice, suggesting that the macrophages and monocytes whose numbers were increased in CD3ϵ^-/-^ mice and CD8^+^T cell-depleted mice had M2-type like characters. We also observed that the monocytes and macrophages whose numbers were increased in CD3ϵ^-/-^ mice and CD8^+^T cell-depleted mice expressed higher levels of tissue-repairing factors such as *Igf1, Areg*, and *Osm* (9, 23, 24); *Igf1* was upregulated in monocyte/macrophage clusters 2, 8, 10, and 13 (**Figure 6C**, left panel), *Osm* was particularly upregulated in monocyte/macrophage cluster 0, 6, 7, and 13 (**Figure 6C**, right panel), and *Areg* was diffusely expressed in the clusters of Ly6c^low^ (**Figure 6C**, middle panel). Upregulation of these genes in mononuclear cells depleted of T cells and B cells was confirmed through conventional RT-PCR analysis (**Figure 6D**).

From RNA velocity analysis and expression pattern, we propose that the absence of CD8^+^T cells creates a niche for monocytes and allows some of these cells to proliferate and differentiate after TAC into Ly6c^low^CCR2^+^ macrophages expressing tissue-repairing growth factors that cause cardioprotective hypertrophy.

## Discussion

The absence of CD4^+^T cells has been reported to alleviate heart failure in the chronic phase of TAC (13). To date, it has been supposed that the absence of CD4^+^T cells suppressed macrophage infiltration and fibrosis following compensatory hypertrophy. In the present study, however, we found that the absence of CD8^+^T cells increases the degree of compensatory hypertrophy but suppresses subsequent heart failure. scRNAseq analysis of myocytes confirmed the protective role of myocytes and their differentiation in the absence of CD8^+^T cells. These data raise a discrepancy between heart hypertrophy and failure; heart hypertrophy in the absence of CD8^+^T cell does not induce more heart failure. We have shown that there are two types of heart hypertrophy; one is compensatory “protective” and the other is “destructive” depending on the time and conditions (16). During early hypertrophy, cardiomyocytes activate mitochondrial translation/metabolism genes, whose expression is correlated with cell size and linked to ERK1/2 and NRF1/2 transcriptional networks. While in late hypertrophy, p53 signaling is activated, followed by mitochondrial inhibition, morphological elongation, and heart failure gene program activation. Thus, we propose that CD8^+^T cells suppress early phase protective heart hypertrophy while CD4^+^T cells suppress late phase destructive hypertrophy which leads heart failure. We propose that CD4^+^ and CD8^+^T cells play different roles in the early and late phases of pressure overload-mediated compensatory cardiomyocyte hypertrophy.

CD8^+^T cells are present in the heart and increased by TAC, but their number is still small. Thus, we suspected that CD8^+^T cells did not directly cause changes in myocytes, but rather affected macrophage phenotype, which has been shown to play important roles in cardiomyocyte hypertrophy and heart failure (25, 26). Macrophage depletion by clodronate liposomes rapidly worsened left ventricular ejection function and led to cardiomyopathy in hypertensive rats (27). In this model, clodronate has been shown to reduce the influx of CD4^+^T cells, yet the molecular mechanism underlying the protective role of macrophages has remained to be clarified until now. Our scRNAseq analysis suggested that these protective effects arise from secreted growth factors and cytokines. Areg produced by Ly6c^low^ macrophages is important for compensatory hypertrophy, and the infusion of Areg^-/-^ Ly6c^low^ macrophages into the myocardium cannot reverse the worsening of heart failure that is caused by the decrease in Ly6c^low^ macrophages (9). IGF-1 has been shown to be required for proper compensatory hypertrophy (23). Oncostatin M is a controversial factor in cardiomyopathy and fibrosis; in myocardial infarction models, however, it causes partial cardiomyocyte dedifferentiation and initially protects damaged myocardium, but long-term induction promotes chronic heart failure (28) and inhibits excessive fibrosis in pressure-overload heart failure models (24).

scRNA sequencing analysis revealed that muscle cells from CD8^+^T cell-depleted or CD3ϵ^-/-^ mice are different in metabolic features from those of WT mice after TAC, and modules M6 and M12 as the gene modules that were most drastically different between WT mice and CD8^+^T cell depleted or CD3ϵ^-/-^ mice (**Figures 3C, D** **)**. ME6 contains genes related to mitochondrial ATP synthesis, and the expression of these genes are lower in CD3ϵ^-/-^ mice and CD8^+^T cell-depleted mice than WT mice. This seems to be contradictory to the notion that cardioprotective hypertrophy links to mitochondrial gene activation (16). We still do not fully understand why reduction of ME6 associates with T cell-depletion, however, we suspected that the decrease in M6 genes linked reduced oxidative stress, and increase of ME12 which contains translation, ATP metabolic process and translation is the genes related to wound healing programs and can overwhelm the effects of the reduction of ME6. Further study is necessary to define the molecular mechanism of protective hypertrophy by T cell depletion.

There are three types of cardiac tissue macrophages: the completely self-renewing TIMD4^+^ LYVE1^+^ group, the MHC-II^+^ group, which is derived from the TIMD4^+^ group, and the CCR2^+^ group, which represents monocyte-derived macrophages (29). In the present study, scRNAseq of tissue macrophages after TAC revealed CCR2-negative, self-renewing clusters, which probably include the TIMD4^+^ LYVE1^+^ group and the MHC-II^+^ group. Within the Ly6c^low^ group of macrophages that proliferates 1 week after TAC, on the other hand, it is the CCR2^+^ macrophages that are affected by the absence of CD8^+^T cells and that produce the growth factors (IGF-1, AREG, OSM) which are supposed to be involved in reduced heart failure. Therefore, although we did not observe strong differences of macrophages in the spleen between WT and CD3ϵ^-/-^ mice, we cannot completely exclude the possibility that there are systemic effects of CD8^+^T cell depletion on CCR2^+^ monocytes. Removal of CD8^+^ macrophages and its effects are important points, however, we could not characterize CD8^+^macrophage and DCs by our scRNAseq analysis. In addition, CD3ϵ gene disruption supposed not to affect macrophages.

The most important question remained is how CD8^+^T cells affect heart-resident and blood-derived macrophages. CD8^+^T cells may directly or indirectly modulate the number and activation of cardiac macrophages. There is a possibility that macrophages are directly killed by CD8^+^T cells. In addition, a heart infarction model recently proposed that M-CSF is produced by CD8^+^T cells (12). M-CSF from cardiomyocytes and infiltrated monocytes has been shown to play important roles in the numbers of heart macrophages and the proportions of M2 phenotypes (30). Systemic GM-CSF may also increase the numbers of cardiac-resident Ly6^low^ macrophages (9). In keeping with this hypothesis, we observed increased M-CSF in the heart in the absence of CD8^+^T cells. The molecular mechanism by which CD8^+^T cells reduce M-CSF expression remains to be clarified. For example, to clarify the role of CD8^+^T cells in the heart, CD8^+^T cells from injured hearts could be transferred into naïve mice. It is of interesting whether CD8^+^T cells generated during TAC induce chronic changes in heart, change in macrophage phenotypes and make disease progressively worse. In addition, TCR analysis of hear resident CD8^+^T cells will facilitate the understanding how CD8^+^T cells modulate heart failure.

In conclusion, this is the first report showing that CD8^+^T cells in the heart are associate with the conversion of resident and infiltrated macrophages into cardioprotective macrophages, leading to heart-destructive cardiomyocyte hypertrophy. We propose that CD8^+^T cells could serve as a novel target in the prevention of heart failure. Identification of antigens that are recognized by heart CD8^+^T cells could uncover the precise mechanism of heart CD8^+^T cell-macrophage interaction. In addition, it is necessary to define the possibility whether long-term immunosuppression would be effective for heart failure.

## Materials and Methods

### Mice

C57BL/6N mice were purchased from CLEA JAPAN. *Cd3*ϵ^-/-^ and OT-I-TCR transgenic mice have been described previously (31). All mice had a C57BL/6 genetic background. Male mice, aged 8 weeks and weighing 20–25 g, were used under cohousing conditions in specific pathogen-free facilities. All animal experiments were approved by the Animal Research Committee and Ethics Committee of Keio University School of Medicine.

### Induction of TAC and Echocardiography

Mice underwent TAC to induce heart failure or a sham operation (32). In TAC, the transverse aorta was constricted with a 7-0 nylon suture across a 27-gauge blunt needle, which was removed after constriction. Transthoracic echocardiography was performed with a Vevo 2100 instrument (VisualSonics) equipped with an MS-400 imaging transducer. Mice were anesthetized during the echocardiographic examination, and heart rate was maintained at 500 to 550 bpm in all mice. M-mode echocardiographic images were obtained from the longitudinal axis to measure left ventricular size and function.

### Cell Preparation and Flow Cytometry (FCM) Analysis

Each mouse was deeply anesthetized and intracardially perfused with 40 mL of ice-cold PBS to exclude blood cells. The heart was dissected, minced with fine scissors, and then enzymatically digested with a cocktail of type II collagenase (Worthington), elastase (Worthington), and DNase I (Roche Diagnostics) for one hour at 37°C with gentle agitation as described elsewhere (33). After digestion, the tissue was triturated and passed through a 70-µm cell strainer. Leukocyte-enriched fractions were isolated through 40% to 65% Percoll (GE Healthcare) density gradient centrifugation as described elsewhere. Cells were removed from the interface and washed with RPMI-1640 supplemented with 2% FBS cell culture medium for further analysis. To prevent the nonspecific binding of antibodies to Fc receptors, isolated cells were first incubated with anti-CD16/32 antibody (2.4G2) at 4°C for five minutes. FCM data were acquired with a FACSCanto II (BD Biosciences) and analyzed with FlowJo 9.9.3 software (FlowJo). All antibodies and reagents are described in **Supplementary Table 1**.

### Histologic Assessment

The heart tissue was fixed in paraformaldehyde overnight, and 5-µm tissue sections were cut and stained with Masson’s trichrome staining or Wheat Germ Agglutinin (WGA)-Rhodamine conjugate (Vector) and DAPI. Measurements and analysis were performed using a BZ-X800 fluorescence microscope and accompanying software (Keyence).

### RNA-Seq and Single-Cell RNA-Seq Analysis of Mouse Cardiomyocytes

Cardiomyocytes were isolated using Langendorff perfusion from the left ventricular free wall 2 weeks after TAC or sham operation. Echocardiography was used to assess whether the heart was appropriately exposed to pressure overload. Mice whose hearts had not been appropriately exposed to pressure overload were excluded from single-cardiomyocyte RNA-seq analysis. Enzymatic dissociation using Langendorff perfusion was performed as described previously (16). To prevent hypercontraction, the cardiomyocytes were resuspended in medium (NaCl 130 mM, KCl 5.4 mM, MgCl2 0.5 mM, NaH2PO4 0.33 mM, D-glucose 22 mM, HEPES 25 mM, FBS 0.2%, pH 7.4) containing a low concentration of calcium (0.1 mM). For bulk RNA-seq of myocardium, cardiomyocytes were lysed and mRNA was extracted using RNeasy Micro Kit (QIAGEN) as described (34). The analysis was performed by GENEWIZ. For scRNAseq, rod-shaped live cardiomyocytes were collected immediately after isolation from two mice with a 0.2–2-µL pipette (sample volume, 0.5 µL) and incubated in lysis buffer. Single-cell cDNA libraries were generated using the Smart-seq2 protocol and the efficiency of reverse transcription was assessed by examining the cycle threshold (Ct) values of control genes (Tnnt2) from quantitative real-time polymerase chain reaction (q-PCR). The following primer sets were used for qPCR: Tnnt2 mRNA forward, TCCTGGCAGAGAGGAGGAAG; Tnnt2 mRNA reverse, TGCAGGTCGAACTTCTCAGC. A Ct value of 25 was set as the threshold. The libraries were sequenced on a HiSeq 2500. RefSeq transcripts (coding and non-coding) were downloaded from the UCSC Genome Browser (http://genome.ucsc.edu). Reads were mapped to the mouse genome (mm9) with the parameters “-g 1 -p 8 mm9—no-coverage-search” using TopHat (35). FPKM was calculated with reads mapped to the nuclear genome (36). We removed the low-quality cardiomyocytes with less than 3,000 detected genes for the downstream analysis. Analysis of the single-cell transcriptome profiles was performed using the R statistical language and environment (16). For weighted co-expression network analysis, all genes expressed at an FPKM value of ≥5 in at least 5 cardiomyocytes were used to construct a signed network using the WGCNA R package (37). Gene ontology analysis was performed using DAVID (38). For pseudotime analysis, Slingshot was used to detect trajectories (39).

### Single-Cell RNA-Seq Analysis of CD45-Positive Mouse Cells

One week after TAC surgery, heart CD45⁺ cells were isolated using an autoMACS Pro Separator by positive selection after Percoll enrichment. To obtain single-cell RNA-seq data, we used an BD Rhapsody system (BD Biosciences) and TAS-Seq as previously reported (40). A single-cell suspension of ~20,000 cells was captured from all isolated cells, without selection, on an array of >200,000 microwells through a limited dilution approach. Beads with oligonucleotide barcodes were added to saturation so that one bead was paired with each cell in a microwell. After exposure to cell lysis buffer, polyadenylated RNA molecules hybridized to the beads. Beads were retrieved into a single tube for reverse transcription and Exonuclease treatment. cDNA amplification was performed by using TAS-Seq approach. Sequencing libraries were prepared by using NEBNext Ultra II FS library prep kit for Illumina (New England Biolabs). The sequencing analysis were performed by ImmunoGeneTeqs, Inc (Chiba, Japan) by a Novaseq 6000 sequencer (Illumina, San Diego, CA, USA) and a Novaseq S4 200 cycles v1.5 kit (Illumina).

### Fastq Data Preprocessing and Demultiplexing of scRNAseq Data of Mouse CD45 Positive Cells

Pair-end fastq files (R1: cell barcode reads, R2: RNA reads) of TAS-Seq data were processed as a previous report. For assignment of each tag to each cell barcode, read counts of each tag in each valid cell barcode, which is defined by the cDNA matrix, were extracted from the tag/cell barcode expression matrix. Unassigned cell barcodes were labeled as “not-detected” cells. Then, a sum of the total read counts of each tag was normalized to the minimum sum count of each tag, and log_2_ fold-change between first most tag counts and second most tag counts within each cell barcode. Each cell barcode was ranked by the fold-change ascending order, and the top 3.28% cells were identified as doublets, which were theoretically detectable doublets calculated by the Poisson’s distribution based on the number of loaded cells, total Rhapsody well number, and the number of tags used. Finally, remained cell barcodes were assigned to the first most counted tags.

### Single-Cell Clustering and Annotation of Mouse CD45 Positive Cells

Clustering of single cells of each dataset was performed by using Seurat v2.3.4 (41) in R 3.6.3. Seurat object for each dataset was created by using CreateSeuratObject function (min.cells=5, min.genes=500). Cells of which mitochondrial gene proportion over 0.4 was filtered out by FilterCells function in Seurat v2.3.4. On the mitochondrial gene proportion vs nGene plot, the nGene low and mitochondrial gene high dead cells are mainly mitochondrial gene proportion above 0.4. The population between 0.25 and 0.4 was also minor, thus we filtered out mitochondrial gene proportion over 0.4. The expression data was normalized by normalizeData function (scale.factor = 1,000,000), and scaled with ScaleData function in Seurat v2.3.4. Read counts of each cell within each dataset were regressing out as a confounding factor within ScaleData function. Highly-variable genes of each dataset were identified using FindVariableGenes function in Seurat v2.3.4. with the following parameters: mean.function = ExpMean, dispersion.function = LogVMR, x.low.cutoff = 0.1, x.high.cutoff = Inf, y.cutoff = 0.5. Then, principal component analysis (PCA) against identified highly-variable genes and projection of PCA onto entire data was performed using RunPCA (number of calculated PCs were 100) and ProjectPCA functions in Seurat v2.3.4. Enrichment of each PC was calculated using JackStraw function (num.replicate = 100), and PCs that were significantly enriched statistically (p ≤ 0.05) were selected for clustering and dimensional reduction analyses. Cell clustering was performed using FindClusters function (resolution = 1.5) in Seurat v2.3.4 against the significant PCs, and dimensional reduction was performed using python wrapper of Fast interpolation-based t-stochastic neighbor embedding (FIt-SNE) (42) v1.2.1 (perplexity = 100, df = 0.9, random_seed=42, max_iter=1000, and all the other parameters were set as defaults) through reticulate package (https://github.com/rstudio/reticulate) in R 3.6.3. Statistically significant marker genes of each identified cluster were identified using parallelized FindMarkers function in Seurat v2.3.4 (test.use=“wilcox”, only.pos=TRUE, min.pct=0.1, logfc.threshold=0.25, adjusted p (Bonferroni correction) ≤ 0.05). Then, each identified cluster was manually annotated by their marker genes that were previously reported as cell subset-defining marker genes, and the lineage marker double-positive cells were annotated as doublets. Next, we further sub-clustered monocytes/macrophages using Seurat v2.3.4 by the similar workflow of whole-cell data, and incorporate their annotation into Seurat object of whole-cell data (resolution = 2, perplexity=50, df = 0.9). Cell subset annotations and their compositions were visualized in 2D FIt-SNE space and stacking plot, respectively. All of the identified marker genes are shown in **Supplementary Table 2**.

### RNA Velocity Analysis of Monocytes and Macrophages

For RNA velocity analysis, TAS-Seq data cDNA reads were mapped to reference genome (build GRCm38_101) by using HISAT2-2.2.1 (43) by the following parameters: -q -p 6 –rna-strandness F –very-sensitive –seed 656565 –reorder –omit-sec-seq –mm. For HISAT2 index build, Corresponded ensembl gtf file was filtered to retain protein-coded RNA, long non-coding RNA, and T cell chain/immunoglobulin chain annotations according to 10X Genomics’s method (https://support.10xgenomics.com/single-cell-gene-expression/software/pipelines/latest/advanced/references#mkgtf). Then, cell barcode information of each read were added to the HISAT2-mapped BAM files, and associated gene annotations were assigned by using featureCounts v2.0.2 (44) by following parameters: -T 2 -Q 0 -s 1 -t gene -g gene_name –primary -M -O –largestOverlap –fraction -R BAM. In featureCounts analysis, “gene” annotation was used for capturing un-spliced RNA information for RNA velocity analysis, and primary annotations were kept. Then, resulted BAM file was split by valid cell barcodes by using nim 1.0.6 and hts-nim v0.2.23. Resulted BAM files were processed into loom files by using velocyto run with -c and -U option, and the loom files were concatenated by loompy.combine function (45). RNA velocity estimation of sub-clustered monocytes/macrophages was performed by using scVelo (46) with reticulate package in R 3.6.3. Briefly, gene filtering and normalization was performed by filter_and_normalize function (min_shared_counts=as.integer (30), min_shared_cells=5), and moments were calculated pp.moments function (n_pcs=32, n_neighbors=30). The number of principal components was determined by the significantly-enriched principal components identified by Jackstraw function of Seurat analysis. RNA velocity was estimated by tl.velocity function with dynamical mode and visualized by pl.velocity_embedding_stream function in 2D FIt-SNE space.

### Quantitative PCR (qPCR)

mRNA was extracted from renal tissues using an RNAiso kit (TaKaRa Bio). Total RNA was reverse-transcribed to cDNA using a high-capacity cDNA reverse transcription kit (Applied Biosystems). qPCR was performed on the cDNA samples using EvaGreen (Bio-Rad). The relative quantification value was expressed as 2^−ΔCt^, in which ΔCt is the difference between the mean Ct value of triplicate measurements and the endogenous Hprt1 control. CD11b^+^ cells (CD45^+^CD11b^+^CD3^-^CD19^-^Ly6g^-^) were sorted on a BD Aria III for qPCR analysis (47).

### *In Vivo* Depletion of CD4^+^ and CD8^+^T Cells and Adoptive Transfer

For CD4^+^ or CD8^+^T cell depletion, mice received an i.p. injection of 100 µg anti-CD4 antibody (GK1.5) or 400 μg anti-CD8 antibody (3.115) on day 0 and every three days until 2 weeks after TAC. For transfer experiments, CD8^+^T cells were isolated from the spleens of WT and OT-I mice using an autoMACS Pro Separator by negative selection (B220^-^CD4^-^CD11b^-^CD11c^-^CD19^-^CD105^-^Gr1^-^NK1.1^-^TCRγδ^-^Ter119^-^). The CD8^+^T cells (1 × 10⁶ cells) were transferred into CD3ϵ^-/-^ mice five days prior to the TAC operation.

### Statistics

Data are expressed as means ± SEM. Statistical significance was determined by one-way ANOVA followed by *post hoc* Tukey’s multiple-comparisons tests to analyze differences among three or more groups, and by unpaired Student’s t-test to analyze differences between two groups. *p* < 0.05 was considered to indicate a significant difference. Statistical analysis was performed using Prism 8.0 (GraphPad Software).

## Data Availability Statement

The datasets presented in this study can be found in online repositories. The names of the repository/repositories and accession number(s) can be found below: NCBI - cardiomyocytes RNAseq DRA012689; cardiomyocytes scRNAseq GSE183024; cardiac CD45 cells scRNAseq GSE183405.

## Ethics Statement

The animal study was reviewed and approved by the Animal Research Committee and Ethics Committee of Keio University School of Medicine.

## Author Contributions

KK and AY designed the research project and performed experiments. MI, SN, SS, MK, SY, TK, MI-K, and HN analyzed the RNAseq data. KK and AY wrote the manuscript with input from all authors. All authors contributed to the article and approved the submitted version.

## Funding

This work was supported by JSPS KAKENHI 17H06175, 21H05044, 19H04817, 21K19382, 21H02719, 21H00432, AMED-CREST JP21gm1110009 and Moonshot JP21zf0127003h0001, AMED-PRIME JP21gm621001010, JP21gm6210012, JPgm6210025, the Yasuda Medical Foundation, Research grant from the Chemo-Sero-Therapeutic Research Institute, the Kishimoto Family Foundation, the Tomizawa Jun-ichi & Keiko Fund of Molecular Biology Society of Japan for Young Scientist, the Mitsubishi Foundation, the Mochida Memorial Foundation for Medical and Pharmaceutical Research, the Takeda Science Foundation, the Uehara Memorial Foundation, the Naito Foundation, the Kanae Foundation, the SENSHIN Medical Research Foundation, the Astellas Foundation for Research on Metabolic Disorders, the Inoue Research Award for Young Scientists, a Life Science Research Award, and Keio Gijuku Academic Developmental Funds.

## Conflict of Interest

The authors declare that the research was conducted in the absence of any commercial or financial relationships that could be construed as a potential conflict of interest.

## Publisher’s Note

All claims expressed in this article are solely those of the authors and do not necessarily represent those of their affiliated organizations, or those of the publisher, the editors and the reviewers. Any product that may be evaluated in this article, or claim that may be made by its manufacturer, is not guaranteed or endorsed by the publisher.

## Supplementary Material

Supplementary Figure 1
**(A)** Gating strategy of analysis of heart CD4^+^T cells and CD8^+^T cells isolated from heart. **(B)** Representative FCM panels for heart CD4^+^T cells and CD8^+^T cells at indicated point. **(C)** Changes of heart weight after TAC. (n=4 in each point) **(D)** Changes in numbers of CD45^+^ cells, CD4^+^T cells and CD8^+^T cells per heart weight after TAC. (n=4 in each point) *p* values vs. day0. **(E)** Gating strategy of analysis of heart CD11b^+^ cells, F4/80^+^ macrophages, and Ly6c^low^cells isolated from heart. **(F)** Changes in numbers of CD11b^+^ cells and F4/80^+^ macrophages per heart weight after TAC. (n=4 in each point) Data points are individual mice in one of two individual experiments. *p* values were determined by two-tailed Student’s t-test. Data are means ± SD. **p* < 0.05, ***p* < 0.01, and ****p* < 0.001.

Supplementary Figure 2
**(A)** FCM panels of cardiac CD4^+^T cells and CD8^+^T cells depletion check performed 1 week after TAC. **(B)** Heart weight 2 weeks after TAC. (n=4-9) **(C)** Transfer schedule (left) and FCM panels of the transfer check performed 1 week after TAC (right). **(D)** Cardiac inflammatory cells 1 week after TAC with transplantation of WT or OT-I-CD8^+^T cells into CD3ϵ^-/-^ mice. (n=4-8) Data points are individual mice in one of two or three individual experiments. *p* values were determined by two-tailed Student’s t-test or one-way analysis of variance (ANOVA). Data are means ± SD. **p* < 0.05, ***p* < 0.01.
