# Supplementary figures and images for: Single-Cell Analysis Revealed the Role of CD8+ Effector T Cells in Preventing Cardioprotective Macrophage Differentiation in the Early Phase of Heart Failure
Source: Front Immunol. 2021 Oct 20;12:763647. doi: 10.3389/fimmu.2021.763647 (PMC8564148; doi:10.3389/fimmu.2021.763647)

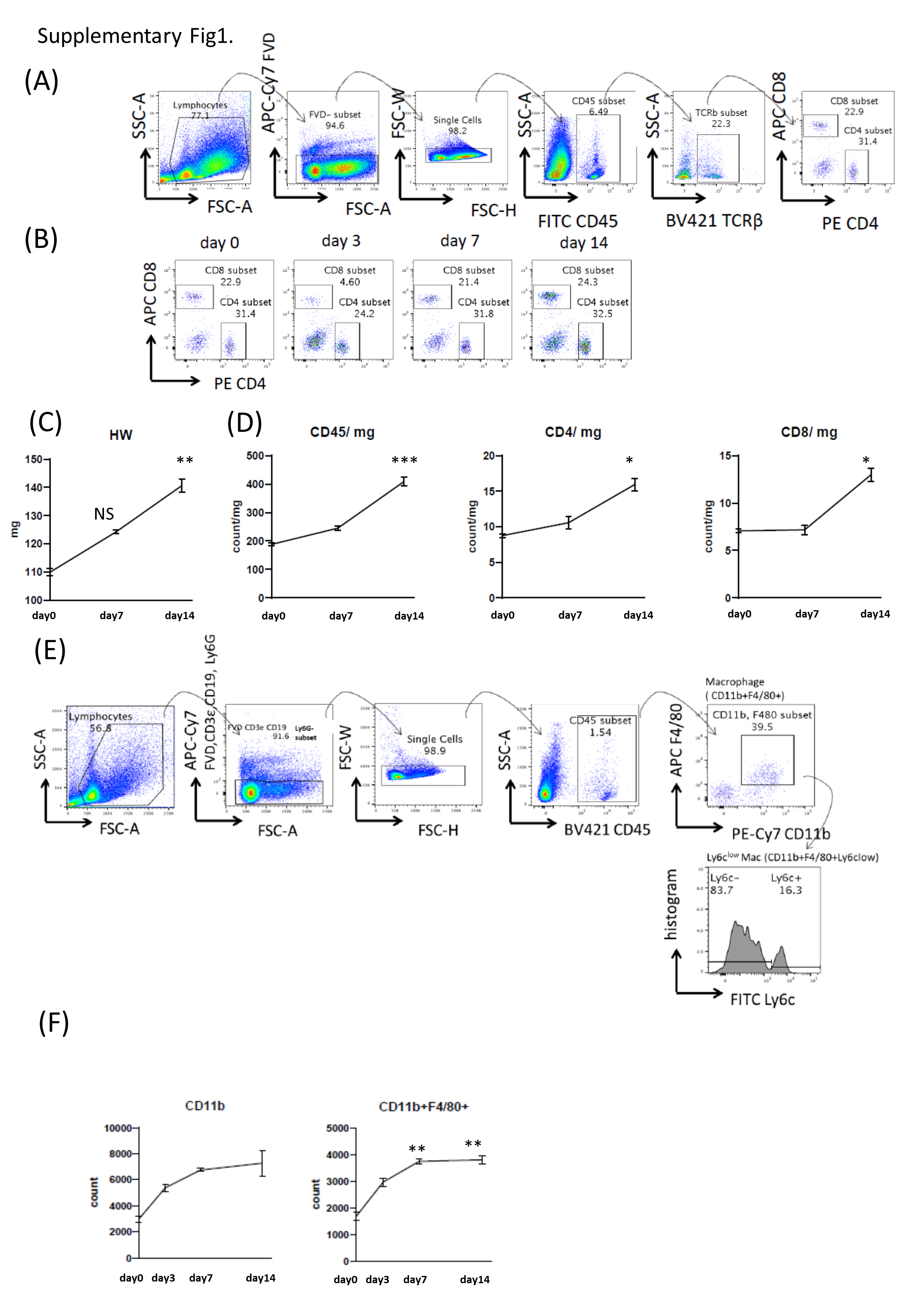

Supplement: Supplementary Figure 1 — (A) Gating strategy of analysis of heart CD4+T cells and CD8+T cells isolated from heart. (B) Representative FCM panels for heart CD4+T cells and CD8+T cells at indicated point. (C) Changes of heart weight after TAC. (n=4 in each point) (D) Changes in numbers of CD45+ cells, CD4+T cells and CD8+T cells per heart weight after TAC. (n=4 in each point) p values vs. day0. (E) Gating strategy of analysis of heart CD11b+ cells, F4/80+ macrophages, and Ly6clowcells isolated from heart. (F) Changes in numbers of CD11b+ cells and F4/80+ macrophages per heart weight after TAC. (n=4 in each point) Data points are individual mice in one of two individual experiments. p values were determined by two-tailed Student’s t-test. Data are means ± SD. *p < 0.05, **p < 0.01, and ***p < 0.001. [file Image_1.tif]

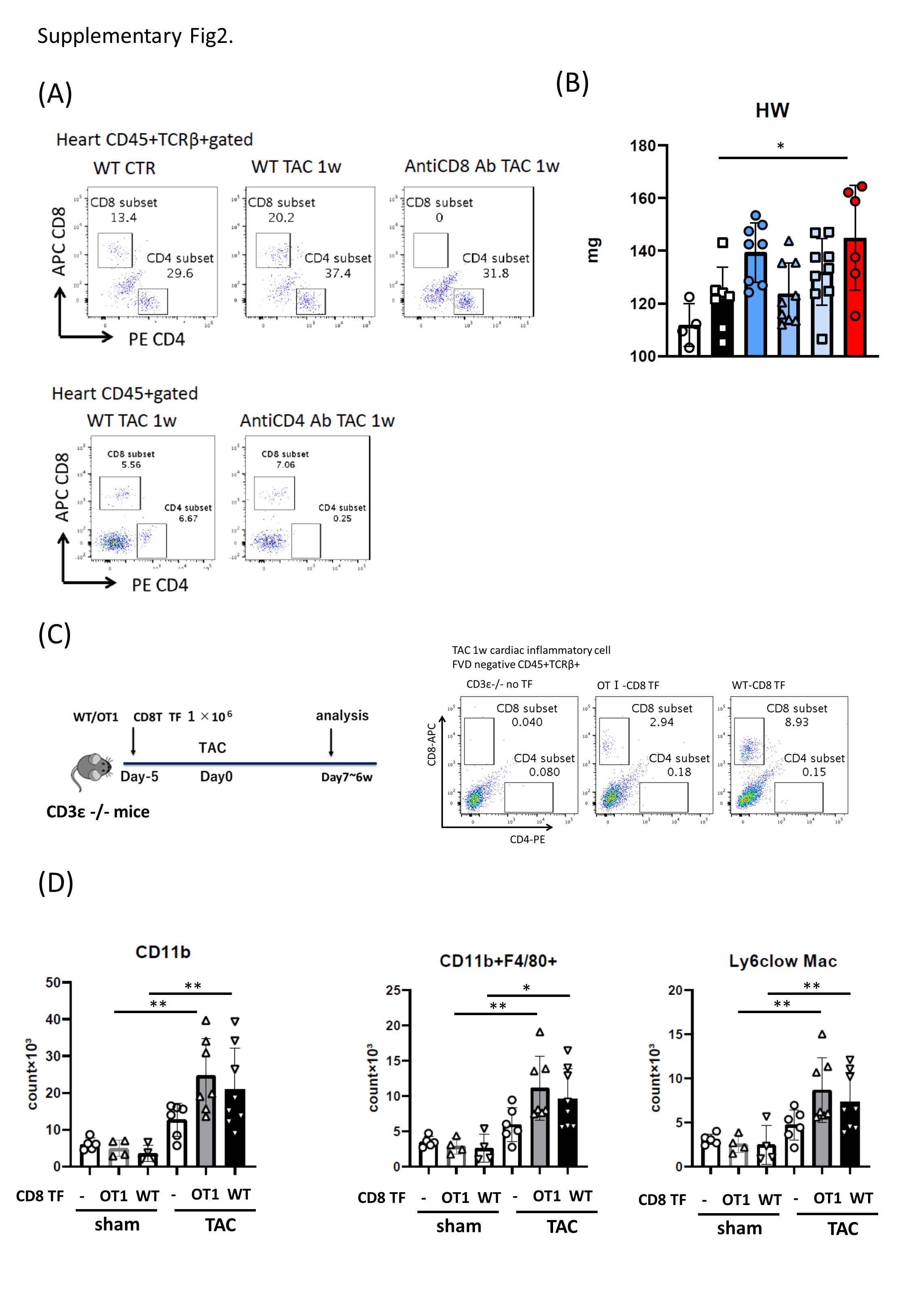

Supplement: Supplementary Figure 2 — (A) FCM panels of cardiac CD4+T cells and CD8+T cells depletion check performed 1 week after TAC. (B) Heart weight 2 weeks after TAC. (n=4-9) (C) Transfer schedule (left) and FCM panels of the transfer check performed 1 week after TAC (right). (D) Cardiac inflammatory cells 1 week after TAC with transplantation of WT or OT-I-CD8+T cells into CD3ϵ-/- mice. (n=4-8) Data points are individual mice in one of two or three individual experiments. p values were determined by two-tailed Student’s t-test or one-way analysis of variance (ANOVA). Data are means ± SD. *p < 0.05, **p < 0.01. [file Image_2.tif]
